# RTS,S/AS01 Malaria Vaccine Efficacy is Not Modified by Seasonal Precipitation: Results from a Phase 3 Randomized Controlled Trial in Malawi

**DOI:** 10.1038/s41598-017-07533-w

**Published:** 2017-08-03

**Authors:** Larry Han, Michael G. Hudgens, Michael E. Emch, Jonathan J. Juliano, Corinna Keeler, Francis Martinson, Portia Kamthunzi, Gerald Tegha, Marc Lievens, Irving F. Hoffman

**Affiliations:** 1 Department of Biostatistics, Gillings School of Global Public Health, University of North Carolina, Chapel Hill, USA; 2 Department of Geography, University of North Carolina, Chapel Hill, USA; 3 Department of Epidemiology, Gillings School of Global Public Health, University of North Carolina, Chapel Hill, USA; 4 School of Medicine, University of North Carolina, Chapel Hill, USA; 5 UNC Project-Malawi, UNC School of Medicine, Lilongwe, Malawi; 6 GlaxoSmithKline Biologicals, Rixensart, Belgium

## Abstract

The World Health Organization has selected Malawi as one of three sites to pilot the roll-out of RTS,S/AS01 in phase 4 trials. As policy discussions for the expanded use of RTS,S/AS01 continue, it will be critical to determine the performance of the vaccine according to seasonal patterns of malaria transmission in regions of Africa. Given waning vaccine efficacy over time, this secondary analysis demonstrates that administering the vaccine to children in the months prior to malaria season could maximize impact of the vaccine. We followed children (5–17 months) and infants (6–12 weeks) assigned to one of three groups: (1) vaccine with four doses; (2) vaccine with three doses; (3) control. The primary endpoint was defined as episodes of clinical malaria. During the 4-years of follow-up, 658 of 1544 (42.6%) children and infants had at least one episode of clinical malaria. With each 1-inch increase in rainfall per month there was an associated increase in the rate of malaria by 12.6% (95% CI 9.6%, 15.6%, P < 0.0001) among children and 15.9% (95% CI 12.8%, 18.9%, P < 0.0001) among infants. There was no evidence of effect modification of vaccine efficacy by precipitation (89% power).

## Introduction

Globally, malaria incidence among at-risk populations fell by 37% between 2000 and 2015, with a corresponding 65% reduction in malaria deaths among children under 5 years of age^1^. Despite substantial financial and developmental resources directed toward the reduction of malaria incidence in Africa, Malawi has not seen a significant decrease in malaria transmission. The entomological inoculation rate (EIR) in Malawi varies depending on the method of sampling and calculation, but previous researchers have estimated a weighted median (25–75 percentile) EIR of 9.1 (1.7, 61.0). This value is similar to Ghana’s weighted median EIR of 7.6 (1.7, 50.4) but is much higher than Kenya’s EIR of 0.2 (0.0, 1.1)^2^. With regard to long-lasting insecticidal-treated nets (LLINs), Malawi has seen an increase in the percentage of households with at least one LLIN from 27% in 2002 to 58% in 2012, with over 18 million LLINs distributed in the decade. Still, this rate is far below the Global Fund’s target of 80% of households with at least one LLIN^3^. Though Malawi’s population is at mesoendemic transmission risk for malaria^4^, it was one of only two (out of 44) malaria-endemic countries that saw an increase in a population-adjusted *Plasmodium falciparum* parasite rate among children aged 2–10 years^5^.

The multi-site, phase 3 trial of RTS,S/AS01, which is the most advanced candidate malaria vaccine, is complete. Across 11 study sites in seven countries in Africa, administration of the vaccine in four doses was shown to reduce clinical malaria by 36.3% (95% confidence interval [CI] 31.8%, 40.5%) in children 5–17 months old and by 25.9% (95% CI 19.9%, 31.5%) in infants 6–12 weeks old, although efficacy varied across sites^6^. The European Medicines Agency has adopted a positive scientific opinion of the vaccine, leading to a recommendation by the WHO’s Strategic Advisory Group of Experts on Immunization (SAGE) and the Malaria Policy Advisory Committee (MPAC) for the implementation of large-scale pilot projects^1^.

In April 2017, the WHO announced that Ghana, Kenya, and Malawi had been selected to participate in the Malaria Vaccine Implementation Program (MVIP) of RTS,S/AS01 beginning in 2018. The three countries were chosen for their high coverage of LLINs, a high malaria burden even after LLIN scale-up, promising malaria and immunization programs, and participation in the Phase 3 trial of RTS,S/AS01^7^. The expected four-year program will provide insights on the feasibility of delivering the vaccine in real-life settings, safety in the context of routine use, and impact on childhood survival rates. Results from the MVIP will be useful before full-scale deployment across the continent. To inform these policy discussions, it will be critical to understand the variation in vaccine efficacy over space and time and within different environmental contexts. It is widely understood that RTS,S/AS01 will not serve as a panacea and that the vaccine must be used in conjunction with other control measures, such as LLINs and prompt and appropriate artemisinin combination therapies. However, implementation of the vaccine may be particularly effective in certain environments, such as areas where malaria transmission is high or seasonal^8^.

Site-specific data that considers the seasonality of vaccine efficacy have been limited. Apart from other programmatic considerations, an understanding of the seasonality of transmission and temporal variations in each pilot location is critical in deploying effective pilot interventions. For example, seasonal malaria chemoprevention is optimally targeted at regions with shorter, more intense malaria transmission seasons and requires accurate timing within that season^9^. Mathematical models have also shown that while absolute vaccine impact would increase with increasing levels of malaria transmission, a higher proportion of averted episodes and high cost-effectiveness may be possible in lower transmission regions^10^. Precipitation is particularly interesting because greater exposure to infected mosquito bites during the peak rainy season may overwhelm vaccine efficacy. We undertook an analysis of the phase 3 trial of RTS,S/AS01 in Lilongwe, Malawi, to evaluate its efficacy against episodes of clinical *Plasmodium falciparum* malaria and its effect modification by seasonal variation in precipitation.

## Methods

### Study Design and Precipitation Data

The details of the study design have been described previously^11–13^. The primary endpoint was defined as development of clinical malaria (fever ≥37.5 °C and *Plasmodium falciparum* parasitemia density >5,000 parasites per microliter) for both age groups^11–13^. Participants were followed passively but parents or legal representatives were told to seek medical care for participants at specified health settings for any illness. Parents or legal representatives of all participants witnessed and provided informed consent at enrollment to the study. Non-literate parents indicated consent by giving a thumbprint in place of a signature. Precipitation data were obtained from the Chitedze Agricultural Center in Lilongwe, Malawi (−13.97S, 33.63E, 3770 ft). The trial protocol was approved by both the institutional review board (IRB) at UNC Chapel Hill and by the Malawi National Health Sciences Research Committee.

### Randomization and Masking

From June 2009 until January 2011, children aged 5–17 months and infants aged 6–12 weeks were recruited in Lilongwe and randomly assigned (1:1:1) by block randomization to one of three groups: (1) a group that received 3 doses of RTS,S/AS01 and a fourth dose 18 months after the third dose (V + 1 group); (2) a group that received the same schedule with a control as fourth dose (V group); (3) a group that received a rabies vaccine (VeroRab, Sanofi-Pasteur) for children 5–17 months of age and a meningococcal serogroup C conjugate vaccine (Menjugate, Novartis) for infants 6–12 weeks of age (C group).

### Procedures

Malaria treatment during the study was done in accordance with Malawi national guidelines and cases were detected by passive surveillance. The Lilongwe vaccine trial catchment area was identified using neighborhood location information from patients who came to the Area 18 Health Center in the previous year. Three different geographic data sources were examined to identify the catchment area. To recruit participants, community-based information sessions were conducted for families with infants and children who met the enrollment age criteria. Flyers were also distributed in the identified communities.

### Statistical Analysis

Analyses of vaccine efficacy against clinical malaria were explored in an intention-to-treat cohort and included all episodes from the time of enrolment until the date on which the last enrolled infant or child completed follow-up.

Vaccine efficacy was defined as the percent reduction in the incidence or hazard rate. Cox proportional-hazards models were used to compare rates of first or only malaria episodes between groups^14^. Monthly precipitation was considered in the proportional-hazards model as a time-varying covariate with a time lag of two months. Interaction terms for vaccination status and precipitation were included to assess effect modification. Cumulative risk of malaria was estimated using the Kaplan-Meier method, and log-rank tests were conducted to compare malaria incidence between randomized groups.

Vaccine efficacy against multiple malaria episodes was estimated using negative binomial regression and three extensions of the Cox regression model for recurrent events – the Andersen-Gill (AG), the Prentice Williams Peterson total time (PWP-TT), and the Prentice Williams Peterson gap time (PWP-GT) models^15^. In the negative binomial regression model, the logarithm of follow-up time was used as an offset. Waning of vaccine efficacy was assessed in the negative binomial regression by examining efficacy in years one through four of the study follow-up period. The AG model considers time at-risk since study enrolment, assumes a common baseline hazard function for all episodes, and includes a single parameter for the vaccine effect^16^. The PWP models are stratified proportional hazards models where all children and infants are included in the first stratum, children and infants with at least one malaria episode are included in the second stratum, and so forth. The PWP-TT model considers time at-risk since study enrolment, whereas the PWP-GT model resets the time index to zero after each malaria episode^16^. PWP models that allow for vaccine effects to vary across malaria episodes were used to estimate vaccine efficacy in each malaria episode (Supplementary Tables 2, 3). The AG, PWP-TT, and PWP-GT model estimates of vaccine efficacy against multiple malaria episodes were adjusted for monthly precipitation, and interaction terms for vaccination status and precipitation were included to assess effect modification. Events occurring within 14 days following a malaria episode that met the primary case definition were excluded to avoid counting the same episode twice^17^. Data were censored at the end of the follow-up period for each participant or at the date of emigration, withdrawal of consent, or death. Data were analyzed using SAS software, version 9.4.

Power to detect effect modification of vaccine efficacy by precipitation was assessed by a simulation study. R software, version 3.3, was used to conduct the power calculation.

### Ethical Statement

The study protocol was approved by the UNC Institutional Review Board and the GlaxoSmithKline Investigational Site at Lilongwe, Malawi. The trial was overseen by a Data Safety Monitoring Board operating under a charter assisted by a Local Safety Monitor. The study was registered with ClinicalTrials.gov, number NCT00866619. The study was first received on March 19, 2009 and last updated on April 24, 2017.

### Data Availability

The data that supports the findings of this study are available from GlaxoSmithKline but restrictions apply to the availability of these data, which were used under license for the current study, and so are not publicly available. Data are however available from the authors upon reasonable request and with permission of GlaxoSmithKline.

## Results

### Study Participants

A total of 1986 participants were screened. Of the 1055 children who were screened, 220 did not meet eligibility criteria, 8 withdrew consent, 9 migrated, and 18 were lost to follow-up; 800 children received the first dose of treatment. Of the 931 infants who were screened, 92 did not meet eligibility criteria, 4 withdrew consent, 7 migrated, and 2 were lost to follow-up; 826 infants received the first dose of treatment. A total of 1544 participants in Lilongwe were in the intention-to-treat cohort and had surveillance data available after the third dose. Among the 760 children, 247 were randomly assigned to the V + 1 group, 263 to the V group, and 250 to the C group. Among the 784 infants, 255 were randomly assigned to the V + 1 group, 257 to the V group, and 272 to the C group.

### LLIN household use

LLIN household use among study participants was higher than in the general population, with LLIN household use among children at 87.8% in the V + 1 group, 82.6% in the V group, and 85.4% in the C group at study month 31. Among infants, LLIN household use was 90.4% in the V + 1 group, 87.1% in the V group, and 90.9% in the C group (Table 1). The pilot study will be important to assess whether the vaccine can be effectively deployed in real-life settings where LLIN usage rates are lower. Baseline characteristics were similar between the treatment groups within each age group (Table 1).

**Table 1:** Demographic characteristics at baseline and follow-up in Malawian children and infants, n = 1544.

Characteristic	Children				Infants			
	V + 1 (n = 247)	V (n = 263)	C (n = 250)	Total (n = 760)	V + 1 (n = 255)	V (n = 257)	C (n = 272)	Total (n = 784)
Follow-up time – mo								
Median	45	45.1	45.5	45.3	33.8	33.9	34.4	34.1
10^th^-90^th^ percentile	20.9–49.6	19.3–49.3	21.4–49.0	19.9–49.2	20.8–40.3	20.5–40.1	25.2–40.5	21.8–40.4
Age at first dose*								
Median	12.1	11.6	11.2	11.7	7.8	7.9	8.1	7.9
10^th^–90^th^ percentile	6.6–16.5	6.8–16.8	6.5–16.3	6.6–16.5	5.2–11.5	5.3–11.4	5.2–11.4	5.2–11.4
Sex-no. (%)								
Female	124 (50.4)	136 (51.5)	119 (47.6)	379 (49.9)	116 (45.5)	123 (47.9)	137 (50.4)	376 (48.0)
Male	122 (49.6)	128 (48.5)	131 (52.4)	381 (50.1)	139 (54.5)	134 (52.1)	135 (49.6)	408 (52.0)
LLIN use at month 31 - no. (%)	159 (87.8)	166 (82.6)	158 (85.4)	483 (85.2)	178 (90.4)	175 (87.1)	199 (90.9)	552 (89.5)

*Ages given in months for children and in weeks for infants.

### Efficacy against the first or only malaria episode

**Figure 1:**
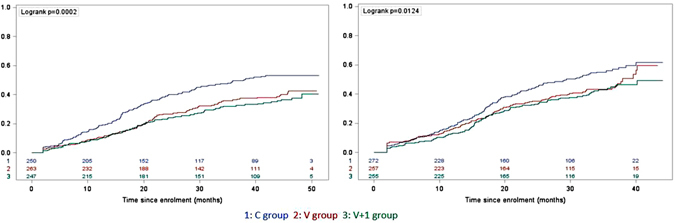
Kaplan-Meier estimates of cumulative risk of first malaria episode among children, n = 760 (left) and infants, n = 784 (right). Y-axis measures the cumulative risk of first malaria episode. Values at each ten-month interval are the number of children or infants remaining at risk.

Overall 658 of 1544 (42.6%) children and infants had at least one episode of clinical malaria. Among children, 299 of 760 (39.3%) developed malaria: 81 of 247 (32.8%) in the V + 1 group, 95 of 263 (36.1%) in the V group, 123 of 250 (49.2%) in the C group. Among infants, 359 of 784 (45.8%) developed malaria: 102 of 255 (40.0%) in the V + 1 group, 112 of 257 (43.6%) in the V group, 145 of 272 (53.3%) in the C group (Table 2). The incidence of first malaria episode was significantly different between treatment arms for children (log-rank test P = 0.0002) and for infants (log-rank test P = 0.01) (Fig. 1a,b). Among children, estimated vaccine efficacies were 41.4% (95% confidence interval (CI) 22.4%, 55.8%, P = 0.0002) for the V + 1 group and 33.8% (95% CI 13.4%, 49.3%, P = 0.003) for the V group. Among infants, estimated vaccine efficacies were 31.2% (95% CI 11.3%, 46.6%, P = 0.004) for the V + 1 group and 23.3% (95% CI 2.8%, 40.0%, P = 0.03) for the V group (Table 2).

**Table 2:** Event rates and efficacy of RTS,S/AS01 vaccine against episodes of *Plasmodium falciparum* clinical malaria.

	V + 1 Group					V Group					C group			
	No. of individuals	No. of events	PYAR*	Event rate	VE (95% CI)	No. of individuals	No. of events	PYAR*	Event rate	VE (95% CI)	No. of individuals	No. of events	PYAR*	Event rate
Children														
First episode	247	81	658.1	0.12	41.4 (22.4, 55.8)	263	95	681.6	0.14	33.8 (13.4, 49.3)	250	123	587.3	0.21
All episodes (NB)**	247	143	811.4	0.18	50.7 (32.6, 64.0)	263	219	853.4	0.26	29.0 (4.7, 47.1)	250	300	839.9	0.36
Infants														
First episode	255	102	540.8	0.19	31.2 (11.3, 46.6)	257	112	536.1	0.21	23.3 (2.8, 40.0)	272	145	537.5	0.27
All episodes (NB)**	255	263	681.7	0.39	31.2 (8.1, 48.5)	257	304	690.2	0.44	20.9 (4.0, 34.9)	272	420	754	0.56

*PYAR: person-years at risk.

**NB: Negative binomial regression.

**Figure 2:**
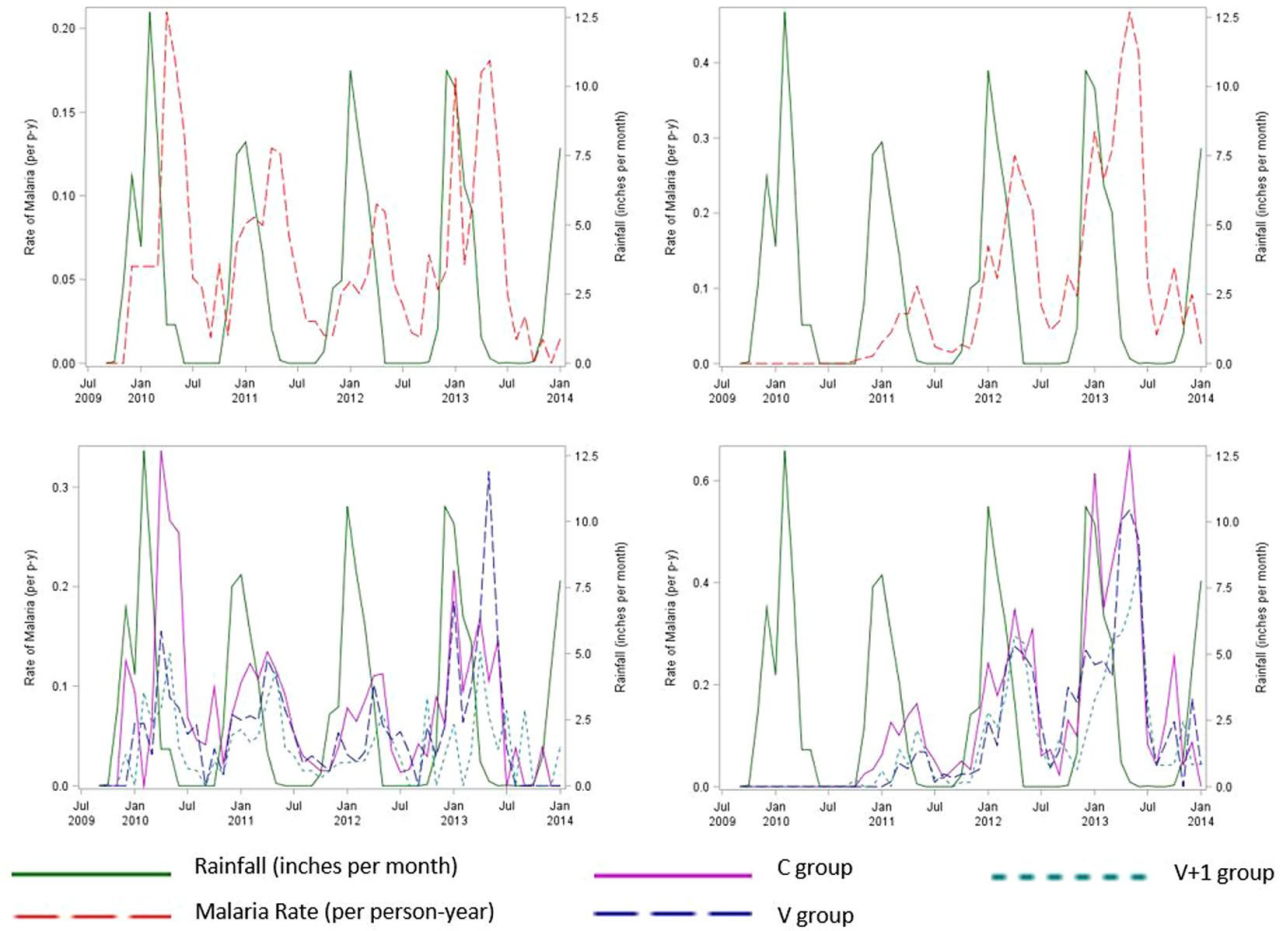
Seasonal precipitation and malaria incidence among children in Lilongwe, Malawi, n = 760 (top left), of treatment groups among children (bottom left), among infants, n = 784 (top right), and of treatment groups among infants (bottom right). Rate of malaria is measured as episodes per person-year (p-y). Note that infants had not yet been enrolled in the trial following the first seasonal rainfall peak in 2010.

Precipitation in the rainy season (November through April) averaged 5.4 inches per month from 2009 through 2014 with a peak mean of 8.2 inches per month in January. Over each study year, malaria incidence was lowest during the period from August through October of the dry season (Fig. 2a–d). Precipitation was significantly and positively associated with malaria incidence, with each 1-inch increase (decrease) in rainfall per month associated with an increase (decrease) in the rate of malaria by 12.6% (95% CI 9.6%, 15.6%, P < 0.0001) among children and by 15.9% (95% CI 12.8%, 18.9%, P < 0.0001) among infants, given a time lag of two months. Cox model interaction terms between the V + 1 group and precipitation (P = 0.78) and between the V group and precipitation (P = 0.85) were non-significant.

### Power to detect effect modification

Power to detect effect modification by precipitation was assessed by a simulation study. Two thousand data sets were simulated as follows: The sample sizes for infants and children allocated to the V + 1 group, V group, and C group matched the sample sizes in the trial. Annual loss to follow-up was 15% and maximum follow-up was four years. Incidence of first malaria episode was 0.27 per person-year for infants in the C group when precipitation was 2.75 inches per month (the average rainfall for the time period under consideration). Vaccine efficacy was 65% when precipitation was less than 2.75 inches per month and vaccine efficacy was 25% otherwise. The average number of malaria episodes in the 2,000 simulated data sets was 654 (similar to the 658 total number of first or only episodes of malaria in the actual data). Effect modification was assessed for each data set by fitting a Cox model and testing for an interaction between vaccination and precipitation. The empirical power to detect effect modification was 89%.

### Efficacy against all episodes

**Figure 3:**
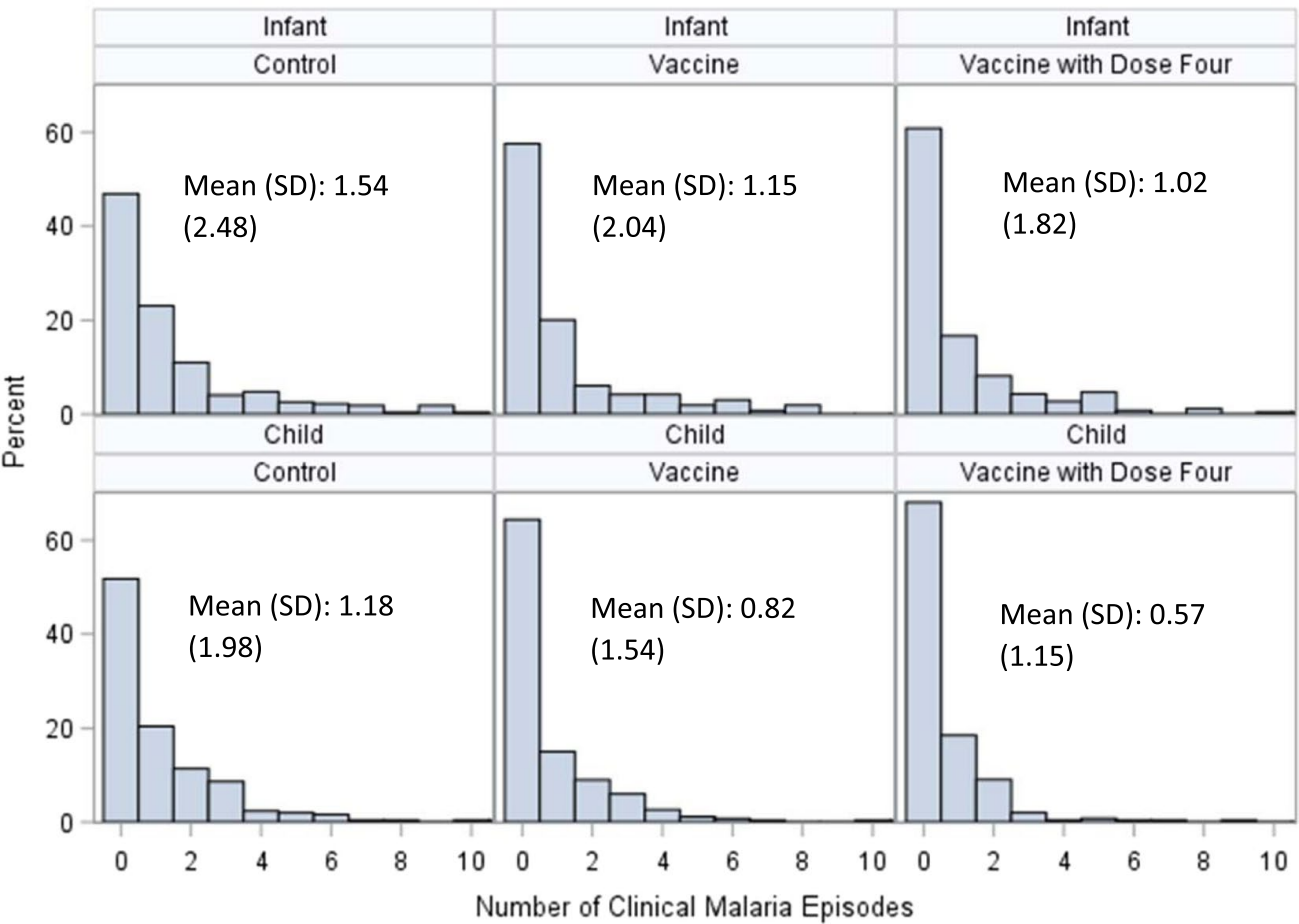
Distribution of malaria episodes among treatment and age groups. SD: Standard deviation.

Multiple malaria episodes were common. From study initiation until study end, there were 143 episodes of clinical malaria that met the primary case definition in children in the V + 1 group, 219 episodes in the V group, and 300 episodes in the C group. In the infant group, there were 263 episodes in the V + 1 group, 304 episodes in the V group, and 420 episodes in the C group (Table 2, Fig. 3).

Survival analysis of all malaria episodes by different methods yielded similar results, although the PWP models with common effects showed lower efficacy estimates compared to the negative binomial and Andersen-Gil models. In the negative binomial regression, among children, vaccine efficacies against multiple episodes were 50.7% (95% CI 32.6%, 64.0%, P < 0.0001) in the V + 1 group and 29.0% (95% CI 4.7%, 47.1%, P = 0.02) in the V group compared to the C group. Among infants, vaccine efficacies against multiple episodes were 31.2% (95% CI 8.1%, 48.5%, P = 0.01) in the V + 1 group and 20.9% (95% CI 4.0%, 34.9%, P = 0.02) in the V group. By the Andersen-Gill model, among children, efficacies against multiple episodes were 49.7% (95% CI 31.6%, 63.5%, P < 0.0001) in the V + 1 group and 29.1% (95% CI 3.8%, 47.7%, P = 0.03) in the V group compared to the C group. Among infants, vaccine efficacies against multiple episodes were 31.1% (95% CI 8.0%, 48.3%, P = 0.01) in the V + 1 group and 27.5% (95% CI 2.6%, 46.1%, P = 0.03) in the V group. By the PWP-TT model with common effects, among children, efficacies against multiple episodes were 33.1% (95% CI 21.2%, 46.0%, P = 0.0002) in the V + 1 group and 14.3% (95% CI 1–3.5%, 29.1%, P = 0.11) in the V group. Among infants, vaccine efficacies against multiple episodes were 13.9% (95% CI −1.5%, 26.9%, P = 0.07) in the V + 1 group and 8.4% (95% CI −7.5%, 22.0%, P = 0.28) in the V group. By the PWP-GT model with common effects, among children, efficacies against multiple episodes were 36.2% (95% CI 21.1%, 48.4%, P < 0.0001) in the V + 1 group and 17.1% (95% CI 0.2%, 31.1%, P = 0.04) in the V group. Among infants, efficacies against multiple episodes were 17.0% (95% CI 2.5%, 29.4%, P = 0.02) in the V + 1 group and 11.8% (95% CI −3.2%, 24.7%, P = 0.12) in the V group (Table 3).

**Table 3:** Comparison of four models for estimating vaccine efficacy against all clinical malaria episodes.

Model*	Children				Infants			
	V + 1 versus C (95% CI)	p-value	V versus C (95% CI)	p-value	V + 1 versus C (95% CI)	p-value	V versus C (95% CI)	p-value
Negative Binomial	50.7% (32.6%, 64.0%)	<0.0001	29.0% (4.7%, 47.1%)	0.02	31.2% (8.1%, 48.5%)	0.01	20.9% (4.0%, 34.9%)	0.01
Andersen-Gill	49.7% (31.6%, 63.5%)	<0.0001	29.1% (3.8%, 47.7)	0.03	31.1% (8.0%, 48.3%)	0.01	27.5% (2.6%, 46.1%)	0.03
PWP Total Time	33.1% (21.2%, 46.0%)	0.0002	14.3% (−3.5%, 29.1%)	0.11	13.9% (−1.5%, 26.9%)	0.07	8.4% (−7.5%, 22.0%)	0.28
PWP Gap Time	36.2% (21.1%, 48.4%)	<0.0001	17.1% (0.2%, 31.1%)	0.04	17.0% (2.5%, 29.4%)	0.02	11.8% (−3.2%, 24.7%)	0.12

*All models except for the negative binomial are adjusted for monthly precipitation.

There was no evidence that vaccine efficacy in children changed during the rainy season, as indicated by the non-significant interaction between treatment group and precipitation in the AG model (P = 0.38 in the V + 1 group; P = 0.73 in the V group), PWP-TT model (P = 0.36 for the V + 1 group; P = 0.59 in the V group), and PWP-GT model (P = 0.47 in the V + 1 group; P = 0.88 in the V group). Neither did vaccine efficacy change for infants during the rainy season, as indicated by the non-significant interaction between treatment group and precipitation in the AG model (P = 0.71 in the V + 1 group; P = 0.51 in the V group), PWP-TT model (P = 0.69 for the V + 1 group; P = 0.28 in the V group), and PWP-GT model (P = 0.75 in the V + 1 group; P = 0.37 in the V group).

### Waning in Vaccine Efficacy

Waning of vaccine efficacy was assessed by estimating efficacy separately for each year of study follow-up. Among children, vaccine efficacy was 70.1% (95% CI 50.8%, 81.8%, P < 0.0001) in the V + 1 group and 55.1% (95% CI 31.6%, 70.4%, P = 0.0002 in the V group in the first year. By the third year children who received RTS,S/AS01 as the booster dose (V + 1 group) were still protected with a vaccine efficacy of 42.1% (95% CI 8.6%, 63.3%, P = 0.02), but those who did not (V group) were no longer protected, with a vaccine efficacy of 25.9% (95% CI −12.5%, 51.1%, P = 0.16). Among infants, vaccine efficacy declined from 63.0% (95% CI 44.3%, 75.4%, P < 0.0001) in the V + 1 group and 41.2% (95% CI 16.8%, 58.5%, P = 0.003) in the first year to 17.5% (95% CI −88.1%, 63.8%, P = 0.65) in the V + 1 group and 11.0% (95% CI −98.7%, 60.1%, P = 0.78) in the V group in the fourth year. By the third year, as in the case of children, infants in the V + 1 group were protected, with a vaccine efficacy of 37.0% (95% CI 9.7%, 56.1%, P = 0.01) while infants in the V group were no longer protected, with a vaccine efficacy of 8.5% (95% CI −26.1%, 33.6%, P = 0.59) (Supplementary Table 1).

## Discussion

While precipitation was associated with the incidence of clinical malaria in a seasonal malaria setting in Malawi, the efficacy of RTS,S/AS01 vaccine was not modified by variations in precipitation patterns. This information will be valuable for roll-out strategies for RTS,S/AS01, particularly in areas with transmission patterns where seasonal malaria chemoprevention is effective. The majority of this burden lies in the Sahel sub-region and a band across sub-Saharan Africa that includes most of Namibia, Zambia, Malawi, and Mozambique, where a combined 39 million children under five are at-risk for malaria^17^. Pilot programs will soon be underway to assess whether routine immunization can deliver the four-dose schedule effectively, whether the vaccine can prevent deaths, and to what extent the safety concerns identified in phase 3 trials are confirmed in larger phase 4 trials^18^. However, for these pilot programs to be successful, they must be implemented in a way that recognizes differences in vaccine performance across a variety of transmission intensity regions. Considering waning vaccine efficacy over time, this study demonstrates that among seasonal transmission regions, implementation of the vaccine should be given differential weighting based on precipitation patterns and its initiation prior to the peak transmission season may maximize impact.

Precipitation was significantly associated with increased malaria incidence after a time lag of two months. This finding suggests that there is a strong, direct association between rainfall and malaria incidence. The intensity of transmission is dependent on the density, biting, and survival rates of the mosquito vector, which are heavily influenced by seasonal temperature and precipitation patterns^18^. A previous study of malaria attack rates in Northern Ghana showed that the risk of infection was lowest during the period of 2–3 months at the end of the dry season^19^. Our model showed that the level of rainfall most strongly predicted malaria incidence after a time lag of two months (60.5 days). This time lag is consistent with previous time series analyses in a holo-endemic area of Ghana and across the East African highlands, which showed that high-resolution precipitation data could directly predict malaria incidence among children less than 15 years of age^20, 21^. This period of time also closely matches the theoretical vector-parasite-host cycle, which includes the growth of the *Anopheles* mosquito from egg to reproductive age, the development of the *Plasmodium falciparum* parasite in the vector from gametocytes to sporozoites, and the incubation period in the human host from infection to onset of malaria symptoms^22^.

Age played an important role in the number of clinical malaria episodes observed, with infants significantly more likely to experience multiple episodes of malaria. Consistent with findings from the phase 3 trial, vaccine efficacies were higher among children compared to infants in the negative binomial model, AG model, and PWP models^6^. This observed difference may be explained in part by differences in naturally acquired immunity and physiological effects between the two age groups^23^. Due to the lower efficacy rates observed among infants, the WHO Strategic Advisory Group of Experts on Immunization and the Malaria Policy Advisory Committee jointly decided that it would not recommend RTS,S vaccination among this younger age group^24^. However, it is likely that continued reductions in malaria exposure will lead to an increased burden among children over five-years old. In a comparison of four mathematical models, enhanced prevention measures and RTS,S vaccination was predicted to cause such an age shift in malaria incidence, disproportionately affecting children of older ages^10^. Pilot implementations should assess the magnitude of the predicted age shift and its impact on severe disease and mortality. Future studies would be needed to establish safety and immunogenicity of the vaccine against older age groups, including adults.

Extensions of the Cox model for estimating vaccine efficacy against all clinical episodes showed significant efficacy for both the V + 1 group and V group. In each instance, the PWP models (total time and gap time) indicated lower vaccine efficacies compared to the negative binomial and Andersen-Gill models (Table 3). This observed difference may be explained in part by the ability of PWP models to estimate RTS,S efficacy for each episode^15^. The negative binomial model is a reasonable choice for estimating an overall effect in disease reduction with time as an offset^6, 25, 26^, but the added value of the PWP models is the ability to accommodate time-dependent variables like precipitation and a more efficient use of data because timing of malaria episodes is considered^16^. The PWP models are especially useful when the effects of covariates such as vaccination and precipitation are different in subsequent episodes, which is likely the case because RTS,S efficacy has been shown to wane over time^6, 16^. However, the PWP models also have limitations. For example, the conditional nature of the models leads to very small, nonrandom risk sets for later episodes^16^, making vaccine efficacy estimates for subsequent episodes imprecise (95% CIs for hazard ratios in Supplementary Tables 2, 3). In light of these methodological differences, it is important that results from extended Cox models are interpreted properly when applied to RTS,S implementation research.

In conclusion, precipitation was significantly associated with increased malaria incidence among children and infants, with a surge in new cases following a time lag of two months. In assessing rates of first malaria episode or all malaria episodes, there was no evidence of effect modification of vaccine efficacy by precipitation. These findings suggest that despite increased malaria incidence in the rainy season, efficacy of RTS,S/AS01 does not change based on time of year. Thus considering waning vaccine efficacy, vaccination prior to peak transmission seasons is likely to maximize impact.

## Electronic supplementary material

Supplementary Tables and Code
