# Supplementary material for: RTS,S/AS01 Malaria Vaccine Efficacy is Not Modified by Seasonal Precipitation: Results from a Phase 3 Randomized Controlled Trial in Malawi
Source: Sci Rep. 2017 Aug 3;7:7200. doi: 10.1038/s41598-017-07533-w (PMC5543056; doi:10.1038/s41598-017-07533-w)
Supplement: Supplementary file 1 — Supplementary Tables and Code [file 41598_2017_7533_MOESM1_ESM.doc]

**SUPPLEMENTARY FILE**

**RTS,S/AS01 Malaria Vaccine Efficacy is Not Modified by Seasonal Precipitation:**

**Results from a Phase 3 Randomized Controlled Trial in Malawi**

Larry Han^1^ BSPH, Prof Michael G. Hudgens^1^ PhD, Prof Michael E. Emch^2,3^ PhD, Prof Jonathan J. Juliano^4^ MD, Corinna Keeler^2^ BA, Dr Francis Martinson^5^ PhD, Dr Portia Kamthunzi^5^ MD, Gerald Tegha^5^ MA, Marc Lievens^6^ MSc, Irving F. Hoffman^4,5^ MPH

^1^ Department of Biostatistics, Gillings School of Global Public Health, University of North Carolina at Chapel Hill

^2^ Department of Geography, University of North Carolina at Chapel Hill

^3^ Department of Epidemiology, Gillings School of Global Public Health, University of North Carolina at Chapel Hill

^4^ School of Medicine, University of North Carolina at Chapel Hill

^5^ UNC Project-Malawi, UNC School of Medicine, Lilongwe, Malawi

^6^ GlaxoSmithKline Biologicals, Rixensart, Belgium

**Correspondence:**

Larry Han

Department of Biostatistics, Gillings School of Global Public Health, UNC Chapel Hill

135 Dauer Drive

3101 McGavran-Greenberg Hall, CB #7420

Chapel Hill, NC 27599-7420

Email: [larryhan320@gmail.com](mailto:larryhan320@gmail.com)

**Keywords:** Malaria vaccine efficacy; Malawi; Precipitation; RTS,S/AS01; Seasonal transmission

**Supplementary Table 1. RTS,S/AS01 vaccine efficacy against episodes of *Plasmodium falciparum* according to year of follow-up**

|  | **V group** | | **V+1 group** | |
| --- | --- | --- | --- | --- |
| **Children** | **VE (95% CI)** | **P-value** | **VE (95% CI)** | **P-value** |
| **Year 1** | 55.1% (31.6%, 70.4%) | 0.0002 | 70.1% (50.8%, 81.8%) | <0.0001 |
| **Year 2** | 29.8% (2.6%, 49.3%) | 0.03 | 41.0% (16.2%, 58.4%) | 0.003 |
| **Year 3** | 25.9% (-12.5%, 51.1%) | 0.16 | 42.1% (8.6%, 63.3%) | 0.02 |
| **Year 4** | -13.5% (-91.7%, 32.8%) | 0.64 | 50.5% (2.3%, 74.9%) | 0.04 |
| **Infants** | **VE (95% CI)** | **P-value** | **VE (95% CI)** | **P-value** |
| **Year 1** | 41.2% (16.8%, 58.5%) | 0.003 | 63.0% (44.3%, 75.4%) | <0.0001 |
| **Year 2** | 28.0% (6.4%, 44.6%) | 0.01 | 21.6% (-1.6%, 39.5%) | 0.07 |
| **Year 3** | 8.5% (-26.1%, 33.6%) | 0.59 | 37.0% (9.7%, 56.1%) | 0.01 |
| **Year 4** | 11.0% (-98.7%, 60.1%) | 0.78 | 17.5% (-88.1%, 63.8%) | 0.65 |

VE: Vaccine Efficacy

CI: Confidence Interval

**Supplementary Table 2. PWP models of children (n=760) with non-common effects* for each malaria episode**

|  | **V group** | | | | **V+1 group** | | | |
| --- | --- | --- | --- | --- | --- | --- | --- | --- |
|  | **Gap time model** | | **Total time model** | | **Gap time model** | | **Total time model** | |
| **Malaria Episode** | **HR (95% CI)** | **P-value** | **HR (95% CI** | **P-value** | **HR (95% CI)** | **P-value** | **HR (95% CI)** | **P-value** |
| **1** | 0.59 (0.45, 0.77) | 0.0001 | 0.59 (0.45, 0.77) | 0.0001 | 0.52 (0.39, 0.69) | <0.0001 | 0.52 (0.39, 0.69) | <0.0001 |
| **2** | 1.13 (0.80, 1.58) | 0.50 | 1.14 (0.81, 1.62) | 0.45 | 0.65 (0.44, 0.98) | 0.04 | 0.73 (0.49, 1.10) | 0.13 |
| **3** | 0.72 (0.45, 1.15) | 0.17 | 0.76 (0.48, 1.21) | 0.25 | 0.62 (0.36, 1.08) | 0.09 | 0.66 (0.38, 1.16) | 0.15 |
| **4** | 1.06 (0.57, 1.95) | 0.86 | 0.99 (0.54, 1.89) | 0.99 | 1.52 (0.71, 3.29) | 0.29 | 1.65 (0.76, 3.59) | 0.21 |
| **5** | 0.86 (0.34, 2.17) | 0.75 | 1.30 (0.49, 3.46) | 0.61 | 1.07 (0.38, 3.02) | 0.91 | 1.00 (0.33, 3.02) | 0.99 |
| **6** | 1.12 (0.38, 3.25) | 0.84 | 1.03 (0.32, 3.30) | 0.96 | 2.49 (0.68, 9.04) | 0.17 | 3.38 (0.66, 3.0) | 0.14 |
| **7** | 3.39 (0.73, 15.74) | 0.12 | 1.00 (0.24, 4.12) | 0.99 | 3.52 (0.73, 16.99) | 0.12 | 1.71 (0.38, 17.17) | 0.49 |
| **8** | 0.36 (0.06, 2.22) | 0.27 | 0.15(0.01, 1.92) | 0.14 | 0.10 (0.01, 1.12) | 0.06 | 0.37 (0.08, 1.68) | 0.20 |
| **9** | 2.05 (0.28, 14.72) | 0.48 | 0.49 (0.05, 4.89) | 0.54 | -- | -- | -- | -- |
| **10** | 1.36 (0.12, 15.32) | 0.81 | 0.99 (0.06, 15.85) | 0.99 | -- | -- | -- | -- |

HR: Hazard Ratio

CI: Confidence Interval

*We do not insist that effects for each malaria episode are equal

Both the PWP total time and gap time models with non-common effects showed significant efficacy in the V+1 group and the V group across both age groups for the first malaria episode. Among children, the PWP gap time model with non-common effects showed significant efficacy in the V+1 group for the second episode as well (P=0.04).

**Supplementary Table 3. PWP models of infants (n=784) with non-common effects* for each malaria episode**

|  | **V group** | | | | **V+1 group** | | | |
| --- | --- | --- | --- | --- | --- | --- | --- | --- |
|  | **Gap time model** | | **Total time model** | | **Gap time model** | | **Total time model** | |
| **Malaria Episode** | **HR (95% CI)** | **P-value** | **HR (95% CI** | **P-value** | **HR (95% CI)** | **P-value** | **HR (95% CI)** | **P-value** |
| **1** | 0.61 (0.47, 0.80) | 0.0003 | 0.60 (0.46, 0.78) | 0.0002 | 0.63 (0.48, 0.83) | 0.0009 | 0.63 (0.48, 0.83) | 0.0012 |
| **2** | 1.05 (0.76, 1.44) | 0.97 | 1.19 (0.86, 1.63) | 0.30 | 0.96 (0.70, 1.33) | 0.82 | 0.98 (0.71, 1.37) | 0.92 |
| **3** | 1.11 (0.76, 1.64) | 0.59 | 1.11 (0.75, 1.63) | 0.61 | 1.19 (0.80, 1.77) | 0.39 | 1.37 (0.92, 2.05) | 0.12 |
| **4** | 0.97 (0.62, 1.51) | 0.89 | 0.97 (0.62, 1.53) | 0.91 | 0.82 (0.52, 1.31) | 0.41 | 0.93 (0.58, 1.50) | 0.77 |
| **5** | 0.74 (0.44, 1.25) | 0.26 | 0.76 (0.44, 1.29) | 0.30 | 1.05 (0.60, 1.82) | 0.87 | 1.27 (0.72, 2.24) | 0.42 |
| **6** | 1.03 (0.54, 1.97) | 0.93 | 1.36 (0.69, 2.67) | 0.37 | 1.61 (0.83, 3.12) | 0.16 | 1.81 (0.92, 3.57) | 0.09 |
| **7** | 0.75 (0.36, 1.56) | 0.44 | 0.78 (0.36, 1.73) | 0.54 | 0.44 (0.19, 1.02) | 0.06 | 0.60 (0.27, 1.33) | 0.21 |
| **8** | 1.00 (0.42, 2.36) | 0.99 | 1.45 (0.53, 3.93) | 0.47 | 0.97 (0.38, 2.45) | 0.95 | 0.85 (0.29, 2.46) | 0.76 |
| **9** | 1.27 (0.44, 3.64) | 0.66 | 1.13 (0.35, 3.63) | 0.84 | 0.70 (0.23, 2.08) | 0.52 | 0.61 (0.19, 1.91) | 0.39 |
| **10** | 0.20 (0.02, 1.58) | 0.13 | 0.28 (0.03, 2.32) | 0.24 | 0.37 (0.08, 1.77) | 0.22 | 0.42 (0.08, 2.22) | 0.31 |

HR: Hazard Ratio

CI: Confidence Interval

*We do not insist that effects for each malaria episode are equal

Both the PWP total time and gap time models with non-common effects showed significant efficacy in the V+1 group and the V group across both age groups for the first malaria episode.

R Code

# Simulation to approximate power to detect effect modification

#

# 17 May 2017

#

# Power was assessed by a simulation study, with 2000 data sets simulated as follows.

# The sample size for infants and children allocated to control and the two vaccines was

# the same as in the paper. Annual loss to follow-up was 15% and maximum follow-up was four years.

# Incidence of first malaria episode was 0.27 per person-year for infants receiving control when

# precipitation was 2.75 inches/month (the average rainfall for the time period under consideration).

# Vaccine efficacy was 65% when precipitation was < 2.75 inches/month and vaccine efficacy was 25% otherwise.

# The average number of malaria cases in the 2000 simulated data sets was 654 (similar to the 658

# total number of first cases of malaria in the actual data) and the power to detect effect modification was 89%.

rm(list=ls())

library(survival)

set.seed(123)

nsims <- 2000 # number of simulations

nc.v1 <- 247

nc.v <- 263

nc.c <- 250

nc <- nc.v1+nc.v+nc.c # 760, total # of children

ni.v1 <- 255

ni.v <- 257

ni.c <- 272

ni <- ni.v1+ni.v+ni.c # 784, total # of infants

n <- ni+nc # 1544, total sample size

n.control <- nc.c + ni.c

n.vaccine <- n-n.control

# 55 months of rain data from Larry Han

rain <- read.table("C://Users//mhudgens//Desktop//H2/LarryHan//rain.txt",header=T)

mean.rain <- mean(rain$Precip)

age <- c(rep(0,ni),rep(1,nc))

vaccine <- c(rep("co",ni.c),rep("v0",ni.v),rep("v1",ni.v1),

rep("co",nc.c),rep("v0",nc.v),rep("v1",nc.v1))

onesim <- function(){

# Follow-up times

# Assume 15% LTFU per year. Admin censoring at 4 yrs

c0 <- rexp(n,.15); c0 <- pmin(c0,4)

# Random start month

start <- sample(1:6,n,replace=T)

# Generate survival times

t0 <- rep(NA,n)

for (ii in 1:n){

precip <- rain$Precip[start[ii]:(start[ii]+48)]

# Monthly hazard

haz <- 0.27/12*exp(

(vaccine[ii]=="v0")*log(.35)+

(vaccine[ii]=="v1")*log(.35)+

(age[ii]==1)*log(.780)+

(precip-mean.rain)*0.12197 +

(vaccine[ii]!="co" & precip>mean.rain)*log(.75/.35) )

chaz <- cumsum(haz) # Cumulative hazard by month

fii <- 1-exp(-chaz) # CDF for individual i up to 48 months

t0[ii] <- length(fii[fii<runif(1)])/12 # Survival time in years

}

# Observed data

x0 <- pmin(t0,c0); delta0 <- 1*(t0<c0)

# Analyze via Cox model

fit <- coxph(Surv(x0,delta0)~tt(start)+vaccine+age+tt(start)*vaccine,

tt = function(x, t,...)

rain$Precip[round(t*12)+x]

)

# Wald test of effect modification. Chi-sq w/ 2 df

wald <- fit$coef[5:6]%*%solve(fit$var[5:6,5:6])%*%fit$coef[5:6]

pvalue <- 1-pchisq(wald,2)

reject <- 1*(pvalue<0.05)

counts <- as.data.frame(table(delta0))

c(reject,counts[2,2])

}

getpower <- function(){

power <- matrix(NA,nsims,2)

for (ii in 1:nsims) power[ii,] <- onesim()

power <- apply(power,2,mean)

print(power)}

getpower()

SAS Code

*****************************************

*TITLE: RTS,S Phase 3 Analysis

*DESCRIPTION: SAS Code for RTS,S Survival Analysis*

----------------------------------------

*LANGUAGE: SAS, Version 9.4

*NAME: Larry Han;

libname han "/folders/myfolders";

options mergenoby=warn nodate nonumber;

*Setting up data for PWP analysis*;

data han.allvis_sep24_1;

set han.allvis_sep24correct;

if malever=0 then delete;

run;

data han.allvis_sep24_2;

retain ID status Visit_Date V2_Date Vlast_Date;

set han.allvis_sep24_1;

if status=0 then delete;

run;

data han.allvis_sep24_3;

set han.allvis_sep24_2;

by ID;

lagvdate=lag(visit_date_sas);

if first.ID=1 then gaptime=visit_date_sas-v2_date_sas;

else gaptime=visit_date_sas-lagvdate;

run;

proc sort data=han.allvis_sep24_3; by ID Visit_date_sas; run;

data han.allvis_sep24_4;

set han.allvis_sep24_3;

Tstop=Visit_date_sas-V2_date_sas;

Tstart=Tstop-Gaptime;

run;

data han.allvis_sep24_5;

set han.allvis_sep24_4;

by ID;

output;

if last.ID then do;

status=0;

Tstart=Tstop;

Tstop=Vlast_date_sas-V2_date_sas;

Gaptime=Tstop-Tstart;

output;

end;

run;

*Create dataset for mal_ever=0, to be merged back later for PWP*;

data han.onevis_mal0;

set han.allvis_sep24correct;

if malever=1 then delete;

run;

data han.onevis_mal0a;

set han.onevis_mal0;

by ID;

if first.id then output;

run;

*Malever=0 dataset is ready to be merged back*;

data han.onevis_mal0b;

set han.onevis_mal0a;

if malever=. then delete;

TStart=0;

TStop=fup_days;

Gaptime=TStop-TStart;

run;

*Merge han.allvis_sep24_5 and han.onevis_mal0b for PWP;

proc sort data=han.allvis_sep24_5; by ID Visit_Date_sas; run;

proc sort data=han.onevis_mal0b; by ID Visit_Date_sas; run;

data han.pwp_27march;

set han.allvis_sep24_5 han.onevis_mal0b;

run;

proc sort data=han.pwp_27march; by ID; run;

proc print data=han.pwp_27march(obs=30); run;

data han.pwp_27march;

set han.pwp_27march;

if gaptime<14 then delete; *refer to literature*;

run;

*Add enumeration variable*;

proc sort data=han.pwp_27march; by ID; run;

data han.pwp_28march;

set han.pwp_27march;

Enum + 1;

by ID;

if first.ID then Enum = 1;

run;

*Merge in vacrand and age group (han.trtage) data*;

proc sort data=han.trtage; by id; run;

proc sort data=han.pwp_28march; by ID; run;

data han.pwp_29march;

merge han.trtage han.pwp_28march;

by ID;

run;

data han.pwp_29march;

set han.pwp_29march;

if status=. then delete;

run;

*Name vaccine variable*;

data han.pwp_30march;

set han.pwp_29march;

if Trt='RTS,S - MCC b' or Trt='iRTS,S - MCC b' then Vaccine='R3C';

else if Trt='Rabies - MCC b' or Trt='iMCC - MCC' then Vaccine='C3C';

else Vaccine='R3R';

run;

*************************************************************************************

*************************************************************************************

*PWP Recurrent clinical malaria case analysis*;

proc phreg data=han.pwp_30march;

class Vaccine(ref='C3C') Age;

model (TStart,TStop)*status(0)=Vaccine Age / ties=efron risklimits;

strata Enum;

run;

proc phreg data=han.pwp_30march;

class Vaccine(ref='C3C') Age;

model (TStart,TStop)*status(0)=Vaccine Age Precipitation rxp1 rxp2/ ties=efron risklimits;

array precip{*} precip1-precip84;

fup_months=round(fup_days/30.5);

precipitation=precip[fup_months-2];

rxp1=0;

rxp2=0;

if Vaccine='R3C' then rxp1=precipitation;

if Vaccine='R3R' then rxp2=precipitation;

strata Enum; *If one does not include the 'Strata' statement, then very different answer.;

run;

*For children*;

proc phreg data=han.pwp_30marchchild;

class Vaccine(ref='C3C');

model (TStart,TStop)*status(0)=Vaccine Precipitation rxp1 rxp2/ ties=efron risklimits;

array precip{*} precip1-precip84;

fup_months=round(fup_days/30.5);

precipitation=precip[fup_months-2];

rxp1=0;

rxp2=0;

if Vaccine='R3C' then rxp1=precipitation;

if Vaccine='R3R' then rxp2=precipitation;

strata Enum; *If one does not include the 'Strata' statement, then very different answer.;

run;

*For infants*;

proc phreg data=han.pwp_30marchinf;

class Vaccine(ref='C3C');

model (TStart,TStop)*status(0)=Vaccine Precipitation rxp1 rxp2/ ties=efron risklimits;

array precip{*} precip1-precip84;

fup_months=round(fup_days/30.5);

precipitation=precip[fup_months-2];

rxp1=0;

rxp2=0;

if Vaccine='R3C' then rxp1=precipitation;

if Vaccine='R3R' then rxp2=precipitation;

strata Enum; *If one does not include the 'Strata' statement, then very different answer.;

run;

*Event rate for all episodes*;

data han.pwp_30march2;

set han.pwp_30march;

by ID;

fup_year=fup_days/365.25;

if first.ID=1 then output;

run;

proc sort data=han.pwp_30march2; by vaccine age; run;

proc means data=han.pwp_30march2 sum N;

by vaccine age;

var fup_year malcount;

run;

*PWP Gap time model*;

proc phreg data=han.pwp_30march;

class Vaccine(ref='C3C') Age;

model Gaptime*status(0)=Vaccine Age Precipitation rxp1 rxp2/ ties=efron risklimits;

array precip{*} precip1-precip84;

fup_months=round(fup_days/30.5);

precipitation=precip[fup_months-2];

rxp1=0;

rxp2=0;

if Vaccine='R3C' then rxp1=precipitation;

if Vaccine='R3R' then rxp2=precipitation;

strata Enum;

run;

*For children*;

proc phreg data=han.pwp_30marchchild;

class Vaccine(ref='C3C');

model Gaptime*status(0)=Vaccine Precipitation rxp1 rxp2/ ties=efron risklimits;

array precip{*} precip1-precip84;

fup_months=round(fup_days/30.5);

precipitation=precip[fup_months-2];

rxp1=0;

rxp2=0;

if Vaccine='R3C' then rxp1=precipitation;

if Vaccine='R3R' then rxp2=precipitation;

strata Enum;

run;

*For infants*;

proc phreg data=han.pwp_30marchinf;

class Vaccine(ref='C3C');

model Gaptime*status(0)=Vaccine Precipitation rxp1 rxp2/ ties=efron risklimits;

array precip{*} precip1-precip84;

fup_months=round(fup_days/30.5);

precipitation=precip[fup_months-2];

rxp1=0;

rxp2=0;

if Vaccine='R3C' then rxp1=precipitation;

if Vaccine='R3R' then rxp2=precipitation;

strata Enum;

run;

*Andersen Gill Model*;

*For children*;

data han.pwp_30marchchild;

set han.pwp_30march;

if age='Inf' then delete;

run;

proc phreg data=han.pwp_30marchchild covs(aggregate);

class Vaccine(ref='C3C');

model (Tstart,Tstop)*status(0)=Vaccine Precipitation rxp1 rxp2/ties=efron risklimits;

id ID;

array precip{*} precip1-precip84;

fup_months=round(fup_days/30.5);

precipitation=precip[fup_months-2];

rxp1=0;

rxp2=0;

if Vaccine='R3C' then rxp1=precipitation;

if Vaccine='R3R' then rxp2=precipitation;

run;

*For infants*;

data han.pwp_30marchinf;

set han.pwp_30march;

if age='Child' then delete;

run;

proc phreg data=han.pwp_30marchinf covs(aggregate);

class Vaccine(ref='C3C');

model (Tstart,Tstop)*status(0)=Vaccine Precipitation rxp1 rxp2/ties=efron risklimits;

id ID;

array precip{*} precip1-precip84;

fup_months=round(fup_days/30.5);

precipitation=precip[fup_months-2];

rxp1=0;

rxp2=0;

if Vaccine='R3C' then rxp1=precipitation;

if Vaccine='R3R' then rxp2=precipitation;

run;

*PWP model with noncommon effects*;

data han.pwp_19July;

set han.pwp_30march;

keep Vaccine Age Tstart Tstop Enum Gender fup_days gaptime ID status Precip1-Precip84;

run;

data han.pwp_19July2(drop=LastStatus);

retain LastStatus;

set han.pwp_19July;

by ID;

if first.ID then LastStatus=1;

if (Status=0 and LastStatus=0) then delete;

LastStatus=Status;

if Vaccine='C3C' then Vac='0';

else if Vaccine='R3C' then Vac='1';

else if Vaccine='R3R' then Vac='2';

run;

title 'PWP Total Time Model with Noncommon Effects';

proc phreg data=han.pwp_19July2;

class Vaccine(ref='C3C') Age;

model (TStart,Tstop)*status(0) = Vac1-Vac10 Vacb1-Vacb10 Age Precipitation rxp1 rxp2/ties=efron risklimits;

id ID;

array precip{*} precip1-precip84;

fup_months=round(fup_days/30.5);

precipitation=precip[fup_months-2];

rxp1=0;

rxp2=0;

if Vaccine='R3C' then rxp1=precipitation;

if Vaccine='R3R' then rxp2=precipitation;

Vac1= 1*(Vaccine='R3C' & Enum=1);

Vacb1= 1*(Vaccine='R3R' & Enum=1);

Vac2= 1 * (Vaccine='R3C' & Enum=2);

Vacb2= 1 * (Vaccine='R3R' & Enum=2);

Vac3=1*(Vaccine='R3C' & Enum=3);

Vacb3= 1 * (Vaccine='R3R' & Enum=3);

Vac4=1*(Vaccine='R3C' & Enum=4);

Vacb4= 1 * (Vaccine='R3R' & Enum=4);

Vac5=1*(Vaccine='R3C' & Enum=5);

Vacb5= 1 * (Vaccine='R3R' & Enum=5);

Vac6=1*(Vaccine='R3C' & Enum=6);

Vacb6= 1 * (Vaccine='R3R' & Enum=6);

Vac7=1*(Vaccine='R3C' & Enum=7);

Vacb7= 1 * (Vaccine='R3R' & Enum=7);

Vac8=1*(Vaccine='R3C' & Enum=8);

Vacb8= 1 * (Vaccine='R3R' & Enum=8);

Vac9=1*(Vaccine='R3C' & Enum=9);

Vacb9= 1 * (Vaccine='R3R' & Enum=9);

Vac10=1*(Vaccine='R3C' & Enum=10);

Vacb10= 1 * (Vaccine='R3R' & Enum=10);

strata Enum;

run;

*Children only*;

data han.pwp_19July2child;

set han.pwp_19July2;

if age='Inf' then delete;

run;

proc phreg data=han.pwp_19July2child;

class Vaccine(ref='C3C');

model (TStart,Tstop)*status(0) = Vac1-Vac10 Vacb1-Vacb10 Precipitation rxp1 rxp2/ties=efron risklimits;

id ID;

array precip{*} precip1-precip84;

fup_months=round(fup_days/30.5);

precipitation=precip[fup_months-2];

rxp1=0;

rxp2=0;

if Vaccine='R3C' then rxp1=precipitation;

if Vaccine='R3R' then rxp2=precipitation;

Vac1= 1*(Vaccine='R3C' & Enum=1);

Vacb1= 1*(Vaccine='R3R' & Enum=1);

Vac2= 1 * (Vaccine='R3C' & Enum=2);

Vacb2= 1 * (Vaccine='R3R' & Enum=2);

Vac3=1*(Vaccine='R3C' & Enum=3);

Vacb3= 1 * (Vaccine='R3R' & Enum=3);

Vac4=1*(Vaccine='R3C' & Enum=4);

Vacb4= 1 * (Vaccine='R3R' & Enum=4);

Vac5=1*(Vaccine='R3C' & Enum=5);

Vacb5= 1 * (Vaccine='R3R' & Enum=5);

Vac6=1*(Vaccine='R3C' & Enum=6);

Vacb6= 1 * (Vaccine='R3R' & Enum=6);

Vac7=1*(Vaccine='R3C' & Enum=7);

Vacb7= 1 * (Vaccine='R3R' & Enum=7);

Vac8=1*(Vaccine='R3C' & Enum=8);

Vacb8= 1 * (Vaccine='R3R' & Enum=8);

Vac9=1*(Vaccine='R3C' & Enum=9);

Vacb9= 1 * (Vaccine='R3R' & Enum=9);

Vac10=1*(Vaccine='R3C' & Enum=10);

Vacb10= 1 * (Vaccine='R3R' & Enum=10);

strata Enum;

run;

*Infants only*;

data han.pwp_19July2inf;

set han.pwp_19July2;

if age='Child' then delete;

run;

proc phreg data=han.pwp_19July2inf;

class Vaccine(ref='C3C');

model (TStart,Tstop)*status(0) = Vac1-Vac10 Vacb1-Vacb10 Precipitation rxp1 rxp2/ties=efron risklimits;

id ID;

array precip{*} precip1-precip84;

fup_months=round(fup_days/30.5);

precipitation=precip[fup_months-2];

rxp1=0;

rxp2=0;

if Vaccine='R3C' then rxp1=precipitation;

if Vaccine='R3R' then rxp2=precipitation;

Vac1= 1*(Vaccine='R3C' & Enum=1);

Vacb1= 1*(Vaccine='R3R' & Enum=1);

Vac2= 1 * (Vaccine='R3C' & Enum=2);

Vacb2= 1 * (Vaccine='R3R' & Enum=2);

Vac3=1*(Vaccine='R3C' & Enum=3);

Vacb3= 1 * (Vaccine='R3R' & Enum=3);

Vac4=1*(Vaccine='R3C' & Enum=4);

Vacb4= 1 * (Vaccine='R3R' & Enum=4);

Vac5=1*(Vaccine='R3C' & Enum=5);

Vacb5= 1 * (Vaccine='R3R' & Enum=5);

Vac6=1*(Vaccine='R3C' & Enum=6);

Vacb6= 1 * (Vaccine='R3R' & Enum=6);

Vac7=1*(Vaccine='R3C' & Enum=7);

Vacb7= 1 * (Vaccine='R3R' & Enum=7);

Vac8=1*(Vaccine='R3C' & Enum=8);

Vacb8= 1 * (Vaccine='R3R' & Enum=8);

Vac9=1*(Vaccine='R3C' & Enum=9);

Vacb9= 1 * (Vaccine='R3R' & Enum=9);

Vac10=1*(Vaccine='R3C' & Enum=10);

Vacb10= 1 * (Vaccine='R3R' & Enum=10);

strata Enum;

run;

*First vs. subsequent episodes*;

proc phreg data=han.pwp_19July2inf;

class Vaccine(ref='C3C');

model (Tstart, Tstop)*status(0) = Vac1 Vacb1 Vacsubsequent Vacbsubsequent Precipitation rxp1 rxp2/ties=efron risklimits;

id ID;

array precip{*} precip1-precip84;

fup_months=round(fup_days/30.5);

precipitation=precip[fup_months-2];

rxp1=0;

rxp2=0;

if Vaccine='R3C' then rxp1=precipitation;

if Vaccine='R3R' then rxp2=precipitation;

Vac1= 1*(Vaccine='R3C' & Enum=1);

Vacb1= 1*(Vaccine='R3R' & Enum=1);

Vacsubsequent= 1 * (Vaccine='R3C' & Enum >= 2);

Vacbsubsequent= 1* (Vaccine='R3R' & Enum >=2);

strata Enum;

run;

title 'PWP Gap Time Model with Noncommon Effects';

proc phreg data=han.pwp_19July2;

class Vaccine(ref='C3C') Age;

model gaptime*status(0) = Vac1-Vac10 Vacb1-Vacb10 Age Precipitation rxp1 rxp2/ties=efron risklimits;

id ID;

array precip{*} precip1-precip84;

fup_months=round(fup_days/30.5);

precipitation=precip[fup_months-2];

rxp1=0;

rxp2=0;

if Vaccine='R3C' then rxp1=precipitation;

if Vaccine='R3R' then rxp2=precipitation;

Vac1= 1*(Vaccine='R3C' & Enum=1);

Vacb1= 1*(Vaccine='R3R' & Enum=1);

Vac2= 1 * (Vaccine='R3C' & Enum=2);

Vacb2= 1 * (Vaccine='R3R' & Enum=2);

Vac3=1*(Vaccine='R3C' & Enum=3);

Vacb3= 1 * (Vaccine='R3R' & Enum=3);

Vac4=1*(Vaccine='R3C' & Enum=4);

Vacb4= 1 * (Vaccine='R3R' & Enum=4);

Vac5=1*(Vaccine='R3C' & Enum=5);

Vacb5= 1 * (Vaccine='R3R' & Enum=5);

Vac6=1*(Vaccine='R3C' & Enum=6);

Vacb6= 1 * (Vaccine='R3R' & Enum=6);

Vac7=1*(Vaccine='R3C' & Enum=7);

Vacb7= 1 * (Vaccine='R3R' & Enum=7);

Vac8=1*(Vaccine='R3C' & Enum=8);

Vacb8= 1 * (Vaccine='R3R' & Enum=8);

Vac9=1*(Vaccine='R3C' & Enum=9);

Vacb9= 1 * (Vaccine='R3R' & Enum=9);

Vac10=1*(Vaccine='R3C' & Enum=10);

Vacb10= 1 * (Vaccine='R3R' & Enum=10);

strata Enum;

run;

*Children*;

proc phreg data=han.pwp_19July2child;

class Vaccine(ref='C3C');

model gaptime*status(0) = Vac1-Vac10 Vacb1-Vacb10 Precipitation rxp1 rxp2/ties=efron risklimits;

id ID;

array precip{*} precip1-precip84;

fup_months=round(fup_days/30.5);

precipitation=precip[fup_months-2];

rxp1=0;

rxp2=0;

if Vaccine='R3C' then rxp1=precipitation;

if Vaccine='R3R' then rxp2=precipitation;

Vac1= 1*(Vaccine='R3C' & Enum=1);

Vacb1= 1*(Vaccine='R3R' & Enum=1);

Vac2= 1 * (Vaccine='R3C' & Enum=2);

Vacb2= 1 * (Vaccine='R3R' & Enum=2);

Vac3=1*(Vaccine='R3C' & Enum=3);

Vacb3= 1 * (Vaccine='R3R' & Enum=3);

Vac4=1*(Vaccine='R3C' & Enum=4);

Vacb4= 1 * (Vaccine='R3R' & Enum=4);

Vac5=1*(Vaccine='R3C' & Enum=5);

Vacb5= 1 * (Vaccine='R3R' & Enum=5);

Vac6=1*(Vaccine='R3C' & Enum=6);

Vacb6= 1 * (Vaccine='R3R' & Enum=6);

Vac7=1*(Vaccine='R3C' & Enum=7);

Vacb7= 1 * (Vaccine='R3R' & Enum=7);

Vac8=1*(Vaccine='R3C' & Enum=8);

Vacb8= 1 * (Vaccine='R3R' & Enum=8);

Vac9=1*(Vaccine='R3C' & Enum=9);

Vacb9= 1 * (Vaccine='R3R' & Enum=9);

Vac10=1*(Vaccine='R3C' & Enum=10);

Vacb10= 1 * (Vaccine='R3R' & Enum=10);

strata Enum;

run;

*First vs. subsequent episodes*;

proc phreg data=han.pwp_19July2child;

class Vaccine(ref='C3C');

model gaptime*status(0) = Vac1 Vacb1 Vacsubsequent Vacbsubsequent Precipitation rxp1 rxp2/ties=efron risklimits;

id ID;

array precip{*} precip1-precip84;

fup_months=round(fup_days/30.5);

precipitation=precip[fup_months-2];

rxp1=0;

rxp2=0;

if Vaccine='R3C' then rxp1=precipitation;

if Vaccine='R3R' then rxp2=precipitation;

Vac1= 1*(Vaccine='R3C' & Enum=1);

Vacb1= 1*(Vaccine='R3R' & Enum=1);

Vacsubsequent= 1 * (Vaccine='R3C' & Enum >= 2);

Vacbsubsequent= 1* (Vaccine='R3R' & Enum >=2);

strata Enum;

run;

*Infants*;

proc phreg data=han.pwp_19July2inf;

class Vaccine(ref='C3C');

model gaptime*status(0) = Vac1-Vac10 Vacb1-Vacb10 Precipitation rxp1 rxp2/ties=efron risklimits;

id ID;

array precip{*} precip1-precip84;

fup_months=round(fup_days/30.5);

precipitation=precip[fup_months-2];

rxp1=0;

rxp2=0;

if Vaccine='R3C' then rxp1=precipitation;

if Vaccine='R3R' then rxp2=precipitation;

Vac1= 1*(Vaccine='R3C' & Enum=1);

Vacb1= 1*(Vaccine='R3R' & Enum=1);

Vac2= 1 * (Vaccine='R3C' & Enum=2);

Vacb2= 1 * (Vaccine='R3R' & Enum=2);

Vac3=1*(Vaccine='R3C' & Enum=3);

Vacb3= 1 * (Vaccine='R3R' & Enum=3);

Vac4=1*(Vaccine='R3C' & Enum=4);

Vacb4= 1 * (Vaccine='R3R' & Enum=4);

Vac5=1*(Vaccine='R3C' & Enum=5);

Vacb5= 1 * (Vaccine='R3R' & Enum=5);

Vac6=1*(Vaccine='R3C' & Enum=6);

Vacb6= 1 * (Vaccine='R3R' & Enum=6);

Vac7=1*(Vaccine='R3C' & Enum=7);

Vacb7= 1 * (Vaccine='R3R' & Enum=7);

Vac8=1*(Vaccine='R3C' & Enum=8);

Vacb8= 1 * (Vaccine='R3R' & Enum=8);

Vac9=1*(Vaccine='R3C' & Enum=9);

Vacb9= 1 * (Vaccine='R3R' & Enum=9);

Vac10=1*(Vaccine='R3C' & Enum=10);

Vacb10= 1 * (Vaccine='R3R' & Enum=10);

strata Enum;

run;

*First vs. subsequent episodes*;

proc phreg data=han.pwp_19July2inf;

class Vaccine(ref='C3C');

model gaptime*status(0) = Vac1 Vacb1 Vacsubsequent Vacbsubsequent Precipitation rxp1 rxp2/ties=efron risklimits;

id ID;

array precip{*} precip1-precip84;

fup_months=round(fup_days/30.5);

precipitation=precip[fup_months-2];

rxp1=0;

rxp2=0;

if Vaccine='R3C' then rxp1=precipitation;

if Vaccine='R3R' then rxp2=precipitation;

Vac1= 1*(Vaccine='R3C' & Enum=1);

Vacb1= 1*(Vaccine='R3R' & Enum=1);

Vacsubsequent= 1 * (Vaccine='R3C' & Enum >= 2);

Vacbsubsequent= 1* (Vaccine='R3R' & Enum >=2);

strata Enum;

run;

*******************************Negative Binomial Reg******************************;

*Simple stats*;

proc freq data=han.allvis_sep25_1;

tables malcount;

run;

proc means data=han.allvis_sep25_1 mean std min max var median sum;

var malcount malever;

run;

*Histogram*;

proc univariate data=han.allvis_sep25_1 noprint;

histogram malcount/midpoints = 0 to 20 by 1 vscale = percent;

run;

*Mal_count by fup_days*;

proc sort data=han.allvis_sep25_1; by id; run;

proc sort data=han.trtage; by ID; run;

data han.scatter_27march;

merge han.allvis_sep25_1 han.trtage;

by ID;

run;

data han.scatter_27march;

set han.scatter_27march;

if gender=" " then delete;

fup_months=fup_days/30.4375;

run;

proc sgplot data=han.scatter_27march;

scatter x=fup_months y=malcount / group=age;

run;

*Need to jitter data due to overplotting*;

data han.agejitter(drop=h);

set han.scatter_27march;

h=1;

jit_fup_months=fup_months+h*(ranuni(1)-0.5);

jit_malcount=malcount+h*(ranuni(1)-0.5);

label jit_fup_months="Follow-up time (months)"

jit_malcount="Number of Clinical Malaria Cases";

run;

proc sgplot data=han.agejitter;

scatter x=jit_fup_months y=jit_malcount /group=age;

run;

proc means data=han.scatter_27march;

by age;

var fup_days malcount;

run;

data han.scatter_vac;

set han.scatter_27march;

if Trt='RTS,S - MCC b' or Trt='iRTS,S - MCC b' then Vaccine='R3C';

else if Trt='Rabies - MCC b' or Trt='iMCC - MCC' then Vaccine='C3C';

else Vaccine='R3R';

fup_months=fup_days/30.4375;

run;

proc print data=han.scatter_vac; run;

proc sgplot data=han.scatter_vac;

scatter x=fup_months y=mal_count / group=Vaccine;

run;

proc sgpanel data=han.scatter_vac;

panelby vaccine /uniscale=row rows=1 columns=3;

scatter x=fup_months y=malcount;

colaxis label="Follow-up time (months)";

rowaxis label="Number of Clinical Malaria Cases";

run;

*Need to jitter data due to overplotting*;

data han.jitter(drop=s);

set han.scatter_vac;

s=1;

jfup_months=fup_months+s*(ranuni(1)-0.5);

jmal_count=mal_count+s*(ranuni(1)-0.5);

label jfup_months="Follow-up time (months)"

jmal_count="Number of Clinical Malaria Cases";

run;

proc sgpanel data=han.jitter;

panelby vaccine/uniscale=row rows=1 columns=3;

scatter x=jfup_months y=jmal_count;

run;

proc sgpanel data=han.scatter_vac noautolegend;

panelby vaccine / uniscale=row rows=1 columns=3;

histogram mal_count;

title " ";

colaxis label='Number of Clinical Malaria Cases';

run;

proc sort data=han.scatter_vac;

by trt;

run;

proc means data=han.scatter_vac median p10 p90;

by trt;

var malcount fup_days fup_months;

run;

proc sort data=han.scatter_vac; by age; run;

proc means data=han.scatter_vac median p10 p90;

by age;

var fup_months;

run;

proc sort data=han.scatter_vac; by vaccine; run;

proc means data=han.scatter_vac sum;

by vaccine age;

var mal_count;

run;

*panel by vaccine and age group*;

data han.scatter_vac;

set han.scatter_vac;

if age='Inf' then age_1='Infant';

else if age='Child' then age_1='Child';

if vaccine='C3C' then Group='Control';

else if vaccine='R3C' then Group='Vaccine';

else if vaccine='R3R' then Group='Vaccine with Dose Four';

run;

data han.scatter_vac;

length Group $ 30;

set han.scatter_vac;

run;

ods graphics on;

proc sgpanel data=han.scatter_vac;

panelby age_1 Group / uniscale=all rows=2 columns=3 sort=(descending) layout=panel

rowheaderpos=left novarname;

histogram malcount;

title " ";

colaxis label='Number of Clinical Malaria Episodes' max=10;

run;

*Stats on han.scatter_vac*;

proc sort data=han.scatter_vac; by age; run;

proc means data=han.scatter_vac median p5 p95;

by age;

var malcount fup_months;

run;

proc sort data=han.scatter_vac; by vaccine; run;

proc means data=han.scatter_vac mean std median p5 p95;

by vaccine age;

var malcount fup_months;

run;

proc freq data=han.scatter_vac;

tables vaccine*age*gender /nocol nopercent;

run;

proc freq data=han.scatter_vac;

tables age*gender /nocol nopercent;

run;

*Neg Bin Regression*;

data han.nb;

set han.scatter_vac;

fup_years=fup_days/365.25;

if fup_days=0 then delete;

ln=log(fup_years);

run;

proc genmod data=han.nb;

class vaccine(ref='C3C') age;

model malcount=vaccine age/ dist=negbin link=log offset=ln;

run;

*Take 1-e^(parameter) to find VE*;

proc genmod data=han.nbchild;

class vaccine(ref='C3C');

model malcount=vaccine/ dist=negbin link=log offset=ln;

run;

proc genmod data=han.nbinf;

class vaccine(ref='C3C');

model malcount=vaccine/ dist=negbin link=log offset=ln;

run;

*Cases averted - calculate 95% CI's for rate differences (VB v. C, V v. C)*;

*Need ID, arm, #cases, follow-up time, and log(fup)*;

* genmod gives rates and rate ratio, get confidence interval on the log scale

and then exponentiate the endpoints*;

data averted_child;

set han.nbchild;

keep vaccine mal_count ID fup_years ln;

run;

data han.averted_inf;

set han.nbinf;

keep vaccine mal_count ID fup_years ln;

run;

proc nlmixed data=averted_inf;

lambda = exp(b0 + b1*(vaccine="R3C") + b2*(vaccine="R3R") + ln);

model mal_count ~ poisson(lambda);

estimate "log IR Vaccine with Booster" b0+b2;

estimate "log IR Vaccine" b0+b1;

estimate "log IR Control" b0;

estimate "IRD C v VB" -exp(b0+b2)+exp(b0);

estimate "IRD C v V" -exp(b0+b1)+exp(b0);

run;

proc nlmixed data=averted_child;

lambda = exp(b0 + b1*(vaccine="R3C") + b2*(vaccine="R3R") + ln);

model mal_count ~ poisson(lambda);

estimate "log IR Vaccine with Booster" b0+b2;

estimate "log IR Vaccine" b0+b1;

estimate "log IR Control" b0;

estimate "IRD C v VB" -exp(b0+b2)+exp(b0);

estimate "IRD C v V" -exp(b0+b1)+exp(b0);

run;

proc genmod data=averted_inf;

class vaccine(ref='C3C');

model mal_count=vaccine/dist=poisson link=log offset=ln noint;

estimate "IR Vaccine" vaccine 1 0 0/exp;

estimate "IR Booster" vaccine 0 1 0/exp;

estimate "IR Control" vaccine 0 0 1/exp;

run;

/* How do results change if NB model fit instead? */

proc genmod data=averted_inf;

class vaccine(ref='C3C');

model mal_count=vaccine/dist=nb link=log offset=ln noint;

estimate "IR Vaccine" vaccine 1 0 0/exp;

estimate "IR Booster" vaccine 0 1 0/exp;

estimate "IR Control" vaccine 0 0 1/exp;

run;

/* Code above yields

control 0.74 (95% CI 0.61, 0.89)

vaccine 0.63 (95% CI 0.51, 0.77)

booster 0.51 (95% CI 0.42, 0.62)

ie slight change in point estimates w/ wider CIs */

/* Can also used nlmixed */

proc nlmixed data=averted_inf;

linp=b0+b1*(vaccine="R3C")+b2*(vaccine="R3R");

mu = exp(linp+ln);

p = 1/(1+mu*k);

model mal_count ~ negbin(1/k,p);

estimate "IR Vaccine with Booster" exp(b0+b2);

estimate "IR Vaccine" exp(b0+b1);

estimate "IR Control" exp(b0);

estimate "IR C v VB" -exp(b0+b2)+exp(b0);

estimate "IR C v V" -exp(b0+b1)+exp(b0);

run;

/* yields same IR estimates as genmod,

slightly different CI b/c of log transformation

control 0.74 (95% CI 0.60, 0.88)

vaccine 0.63 (95% CI 0.50, 0.75)

booster 0.51 (95% CI 0.41, 0.61)

Code above also gives NB model IR difference estimates:

c vs vb 0.22 (95% CI 0.05, 0.40)

c vs v 0.11 (95% CI -0.08, 0.30)

Compare w/ Poisson IR difference estimates in paper:

"The estimated number of averted malaria episodes per

100 infants was 12�8 (95 CI 6�5, 25�1) episodes per year

in the vaccine group and 22�7 (95% CI 15�5, 32�5)

episodes per year in the vaccine with booster group,

as compared to the control group."

Take home: NB model IR difference estimates similar, CIs wider

*/

proc nlmixed data=averted_child;

linp=b0+b1*(vaccine="R3C")+b2*(vaccine="R3R");

mu = exp(linp+ln);

p = 1/(1+mu*k);

model mal_count ~ negbin(1/k,p);

estimate "IR Vaccine with Booster" exp(b0+b2);

estimate "IR Vaccine" exp(b0+b1);

estimate "IR Control" exp(b0);

estimate "IR C v VB" -exp(b0+b2)+exp(b0);

estimate "IR C v V" -exp(b0+b1)+exp(b0);

run;

proc univariate data=averted_inf;

var mal_count;

run;

*Likelihood ratio test for overdispersion (-2Loglikelihood) between Poisson and NegBin*;

proc genmod data=han.nb;

class vaccine(ref='C3C') age;

model mal_count=vaccine age/ dist=poisson link=log offset=ln;

run;

********************Cox Regression************************;

proc sort data=han.allvis_sep25_1; by ID; run;

proc sort data=han.trtage; by ID; run;

data han.cox;

merge han.allvis_sep25_1 han.trtage;

by ID;

run;

data han.cox;

set han.cox;

if birthdate=. then delete;

if Trt='RTS,S - MCC b' or Trt='iRTS,S - MCC b' then Vaccine='R3C';

else if Trt='Rabies - MCC b' or Trt='iMCC - MCC' then Vaccine='C3C';

else Vaccine='R3R';

dur_months=dur/30.4375;

run;

proc phreg data=han.cox;

class vaccine(ref='C3C') age;

model dur*malever(0)=vaccine age precipitation rxp1 rxp2 / ties=efron risklimits;

array precip{*} precip1-precip84;

tempdate=dur+v2_date_sas;

diffdays=tempdate-mdy(1,1,2009);

diffmonths=round(diffdays/30.5);

precipitation=precip[diffmonths-2];

rxp1=0;

rxp2=0;

if vaccine='R3C' then rxp1=precipitation;

if vaccine='R3R' then rxp2=precipitation;

run;

*Infants only*;

proc phreg data=han.infcox;

class vaccine(ref='C3C');

model dur*malever(0)=vaccine precipitation/ ties=efron risklimits;

array precip{*} precip1-precip84;

tempdate=dur+v2_date_sas;

diffdays=tempdate-mdy(1,1,2009);

diffmonths=round(diffdays/30.5);

precipitation=precip[diffmonths-2];

rxp1=0;

rxp2=0;

if vaccine='R3C' then rxp1=precipitation;

if vaccine='R3R' then rxp2=precipitation;

run;

*Children only*;

proc phreg data=han.childcox;

class vaccine(ref='C3C');

model dur*malever(0)=vaccine precipitation/ ties=efron risklimits;

array precip{*} precip1-precip84;

tempdate=dur+v2_date_sas;

diffdays=tempdate-mdy(1,1,2009);

diffmonths=round(diffdays/30.5);

precipitation=precip[diffmonths-2];

rxp1=0;

rxp2=0;

if vaccine='R3C' then rxp1=precipitation;

if vaccine='R3R' then rxp2=precipitation;

run;

ods graphics on;

ods select survivalplot(persist) failureplot(persist);;

proc lifetest data=han.infcox plots=survival (failure nocensor test atrisk(maxlen=20) cl);

time dur_months*malever(0);

strata Group;

label dur_months="Time since randomization (months)";

run;

proc lifetest data=han.childcox plots=survival (failure nocensor test atrisk(maxlen=20) cl);

time dur_months*malever(0);

strata Group;

label dur_months="Time until first malaria case (months)";

run;

*********************************;

data han.cox;

set han.cox;

if Vaccine='C3C' then Group='Control';

else if Vaccine='R3C' then Group='Vaccine';

else if Vaccine='R3R' then Group='Vaccine with Dose Four';

if dur=0 then delete;

if age=� � then delete;

run;

data han.cox;

length Group $ 30;

set han.cox;

run;

proc lifetest data=han.cox plots=survival (failure nocensor test atrisk(maxlen=20));

time dur_months*malever(0);

strata Group;

label dur_months="Time since randomization (months)";

run;

*Stratify by age group*;

data han.infcox;

set han.cox;

if age="Child" then delete;

run;

proc lifetest data=han.infcox plots=survival (failure nocensor test atrisk(maxlen=20));

time dur_months*malever(0);

strata Group;

label dur_months="Time since enrolment (months)";

run;

data han.childcox;

set han.cox;

if age="Inf" or age=" " then delete;

run;

proc lifetest data=han.childcox plots=survival (failure nocensor test atrisk(maxlen=20));

time dur_months*malever(0);

strata Group;

label dur_months="Time since enrolment (months)";

run;

ods graphics off;

*Use for Table 1 gender, age group, and vaccine distribution*;

proc freq data=han.cox2;

table age*vaccine*gender;

run;

proc sort data=han.cox2; by vaccine age; run;

proc means data=han.cox2 sum N;

by vaccine age;

var dur_year malever;

run;

*************Time Series***********;

*All malaria cases*;

proc import out=han.Timeser_31March

datafile="/folders/myfolders/Timeseries_30March.xlsx" dbms=xlsx replace;

getnames=yes;

run;

*Plot malaria count from each treatment group*;

ods graphics on;

proc sgplot data=han.timeser_31March;

series x=my y=precipitation / y2axis lineattrs=(color=green pattern=dash);

series x=my y=r3r / lineattrs=(color=orange pattern=longdash);

series x=my y=r3c / lineattrs=(color=purple);

series x=my y=c3c / lineattrs=(color=blue);

xaxis label = 'Month';

yaxis label = 'Count of Malaria';

y2axis label = 'Rainfall (inches per month)';

run;

*Plot simple precipitation*;

proc sgplot data=han.timeser_31March;

series x=my y=precipitation;

run;

*Plot total malaria rate by precipitation*;

proc sgplot data=han.timeser_31March;

series x=my y=precipitation/ y2axis lineattrs=(color=darkgreen);

series x=my y=totalrate /lineattrs=(color=red pattern=mediumdash);

xaxis label = 'Month';

yaxis label = 'Rate of Malaria (per person-year)';

y2axis label = 'Rainfall (inches per month)';

run;

*Plot malaria rate by treatment group by precipitation*;

proc import out=han.Timeser_31March

datafile="/folders/myfolders/Timeseries_30March.xlsx" dbms=xlsx replace;

getnames=yes;

run;

data han.timeser_31March;

set han.timeser_31March;

label precipitation='Rainfall (inches per month)'

c3crate='Control Group'

r3crate='Vaccine Group'

r3rrate='Vaccine with Dose Four'

c3cchildrate='Control'

c3cinfrate='Control'

r3cchildrate='Vaccine'

r3cinfrate='Vaccine'

r3rchildrate='Vaccine with Dose Four'

r3rinfrate='Vaccine with Dose Four'

childrate='Malaria Rate (per p-y)'

infrate='Malaria Rate (per p-y)';

run;

*Combined age groups*;

proc sgplot data=han.timeser_31March;

series x=my y=precipitation/ y2axis lineattrs=(color=darkgreen pattern=solid);

series x=my y=c3crate /lineattrs=(color=darkpurple pattern=solid);

series x=my y=r3crate /lineattrs=(color=darkblue pattern=mediumdash);

series x=my y=r3rrate /lineattrs=(color=teal pattern=shortdash);

xaxis label = 'Month';

yaxis label = 'Rate of Malaria (per p-y)';

y2axis label = 'Rainfall (inches per month)';

run;

*Children*;

proc sgplot data=han.timeser_31March;

series x=my y=precipitation/ y2axis lineattrs=(color=darkgreen pattern=solid);

series x=my y=c3cchildrate /lineattrs=(color=darkpurple pattern=solid);

series x=my y=r3cchildrate /lineattrs=(color=darkblue pattern=mediumdash);

series x=my y=r3rchildrate /lineattrs=(color=teal pattern=shortdash);

xaxis label = 'Month';

yaxis label = 'Rate of Malaria (per person-year)';

y2axis label = 'Rainfall (inches per month)';

run;

proc sgplot data=han.timeser_31March;

series x=my y=precipitation/ y2axis lineattrs=(color=darkgreen);

series x=my y=childrate /lineattrs=(color=red pattern=mediumdash);

xaxis label = 'Month';

yaxis label = 'Rate of Malaria (per person-year)';

y2axis label = 'Rainfall (inches per month)';

run;

*Infants*;

proc sgplot data=han.timeser_31March;

series x=my y=precipitation/ y2axis lineattrs=(color=darkgreen pattern=solid);

series x=my y=c3cinfrate /lineattrs=(color=darkpurple pattern=solid);

series x=my y=r3cinfrate /lineattrs=(color=darkblue pattern=mediumdash);

series x=my y=r3rinfrate /lineattrs=(color=teal pattern=shortdash);

xaxis label = 'Month';

yaxis label = 'Rate of Malaria (per p-y)';

y2axis label = 'Rainfall (inches per month)';

run;

proc sgplot data=han.timeser_31March;

series x=my y=precipitation/ y2axis lineattrs=(color=darkgreen);

series x=my y=infrate /lineattrs=(color=red pattern=mediumdash);

xaxis label = 'Month';

yaxis label = 'Rate of Malaria (per p-y)';

y2axis label = 'Rainfall (inches per month)';

run;

*********************************************************************************;

*Waning VE*;

*Year 1 Child*;

proc import out=han.waning1child

datafile="/folders/myfolders/VE_Waning" dbms=xlsx replace;

sheet='Year1';

getnames=yes;

run;

proc genmod data=han.waning1child;

class vaccine(ref='C3C');

model malcount=vaccine/ dist=negbin link=log;

run;

*Year 2 Child*;

proc import out=han.waning2child

datafile="/folders/myfolders/VE_Waning" dbms=xlsx replace;

sheet='Year2';

getnames=yes;

run;

proc genmod data=han.waning2child;

class vaccine(ref='C3C');

model malcount=vaccine/ dist=negbin link=log;

run;

*Year 3 Child*;

proc import out=han.waning3child

datafile="/folders/myfolders/VE_Waning" dbms=xlsx replace;

sheet='Year3';

getnames=yes;

run;

proc genmod data=han.waning3child;

class vaccine(ref='C3C');

model malcount=vaccine/ dist=negbin link=log;

run;

*Year 4 Child*;

proc import out=han.waning4child

datafile="/folders/myfolders/VE_Waning" dbms=xlsx replace;

sheet='Year4';

getnames=yes;

run;

proc genmod data=han.waning4child;

class vaccine(ref='C3C');

model malcount=vaccine/ dist=negbin link=log;

run;

*Year 1 Infant*;

proc import out=han.waning1inf

datafile="/folders/myfolders/VE_Waning" dbms=xlsx replace;

sheet='y1inf';

getnames=yes;

run;

proc genmod data=han.waning1inf;

class vaccine(ref='C3C');

model malcount=vaccine/ dist=negbin link=log;

run;

*Year 2 Infant*;

proc import out=han.waning2inf

datafile="/folders/myfolders/VE_Waning" dbms=xlsx replace;

sheet='y2inf';

getnames=yes;

run;

proc genmod data=han.waning2inf;

class vaccine(ref='C3C');

model malcount=vaccine/ dist=negbin link=log;

run;

*Year 3 Infant*;

proc import out=han.waning3inf

datafile="/folders/myfolders/VE_Waning" dbms=xlsx replace;

sheet='y3inf';

getnames=yes;

run;

proc genmod data=han.waning1child;

class vaccine(ref='C3C');

model malcount=vaccine/ dist=negbin link=log;

run;

*Year 4 Infant*;

proc import out=han.waning4inf

datafile="/folders/myfolders/VE_Waning" dbms=xlsx replace;

sheet='y4inf';

getnames=yes;

run;

proc genmod data=han.waning4inf;

class vaccine(ref='C3C');

model malcount=vaccine/ dist=negbin link=log;

run;
